# H3N2 Influenza Virus Transmission from Swine to Turkeys, United States

**DOI:** 10.3201/eid1012.040581

**Published:** 2004-12

**Authors:** Young K. Choi, Jee H. Lee, Gene Erickson, Sagar M. Goyal, Han S. Joo, Robert G. Webster, Richard J. Webby

**Affiliations:** * Chungbuk National University, Cheongju, Republic of Korea;; † University of Minnesota, St. Paul, Minnesota, USA;; ‡ North Carolina Department of Agriculture and Consumer Services, Raleigh, North Carolina, USA;; § St. Jude Children's Research Hospital, Memphis, Tennessee, USA

**Keywords:** turkey, influenza virus, H3N2, interspecies transmission, research

## Abstract

In 1998, a novel H3N2 reassortant virus emerged in the United States swine population. We report the interspecies transmission of this virus to turkeys in two geographically distant farms in the United States in 2003. This event is of concern, considering the reassortment capacity of this virus and the susceptibility of turkey to infection by avian influenza viruses. Two H3N2 isolates, A/turkey/NC/16108/03 and A/turkey/MN/764/03, had 98.0% to 99.9% nucleotide sequence identity to each other in all eight gene segments. All protein components of the turkey isolates had 97% to 98% sequence identity to swine H3N2 viruses, thus demonstrating interspecies transmission from pigs to turkeys. The turkey isolates were better adapted to avian hosts than were their closest swine counterparts, which suggests that the viruses had already begun to evolve in the new host. The isolation of swine-like H3N2 influenza viruses from turkeys raises new concerns for the generation of novel viruses that could affect humans.

## Abstract 2

Swinelike H3N2 influenza viruses were isolated from two geographically distinct turkey farms in the United States.

Influenza A virus is a highly infectious pathogen of a limited number of birds and mammals. Individual viruses are generally host-specific and are not readily transmitted between species. The species barrier reflects, at least in part, the different receptor preferences of mammalian and avian viruses. Researchers have suggested that human tracheal epithelial cells lack receptors for the attachment of avian influenza viruses and that avian tracheal epithelial cells lack the appropriate receptors for human viruses (*1*). Pigs, however, possess receptors for both avian and mammalian viruses and are postulated to be the host in which influenza viruses of different origins can genetically reassort (*2**–**4*).

Currently, three subtypes (H1N1, H1N2, and H3N2) of influenza virus are commonly found in pigs worldwide. Depending on the location, these viruses are derived from mammalian viruses, avian viruses, or reassortants of the two (*5*). Until 1998, the classic H1N1 lineage was the only influenza virus circulating widely in the swine population in the United States (*6*). In 1998, H3N2 triple reassortants with genes derived from human (HA, NA, and PB1), swine (M, NS, and NP), and avian viruses (PA and PB2) were first isolated in the United States; they have since become endemic in swine populations (*7**–**9*). These viruses underwent further reassortment to create additional H3N2 viruses isolated from pigs (*8*), as well as H1N2 viruses isolated from pigs (*10**–**12*), turkey (*13*), and wild duck (*14*); this finding demonstrates that viruses containing this gene combination can cross the species barrier. We describe the isolation and characterization of H3N2 influenza viruses from domestic turkeys in the United States. Genetic and serologic characterization showed that these viruses have high homology to each other and to swine influenza viruses recently circulating among pigs in North America.

## Materials and Methods

### Virus Isolation

In 2003, influenza viruses were isolated from two geographically separate turkey farms: one in the Midwest (in February) and one in the eastern United States (in March). We isolated influenza viruses from each farm by using Madin-Darby canine kidney (MDCK) cells supplemented with 1 mg/mL L-(tosylamido-2-phenyl) ethyl chloromethyl ketone (TPCK)-treated trypsin. Briefly, the samples were add to monolayers of MDCK cells and incubated for 1 h at 37°C to allow viral adsorption to the cells. The inoculum was decanted, Eagle's minimum essential medium supplemented with 0.2% bovine serum albumin was added, and monolayers were incubated for 3–5 days at 37°C. After cytopathic effects appeared, influenza virus was confirmed by using hemagglutination of chicken erythrocytes and reverse transcription–polymerase chain reaction (RT-PCR) against the HA gene as previously described (*10*).

### Antigenic Analysis

To examine the antigenic relations between the newly isolated viruses and other avian influenza viruses (i.e., their serologic cross-reactivity), we conducted HI assays with a panel of reference antisera against all 15 HA subtypes and swine antisera to recent swine H3N2 viruses; A/Sw/NC/39615/01, A/Sw/MN/23062/02, A/Sw/TX/46710-35/02, A/Sw/TX/46710-37/03, A/Sw/NC/5854/02, and A/Sw/MO/22583/02. To determine the extent of turkey-to-turkey transmission on infected farms, we collected serum from 12 apparently healthy birds in one infected flock and used the HI assay (*15*) to test for antibodies to influenza virus surface glycoproteins.

### DNA Sequencing

Viral RNA was extracted from supernatants of culture of infected cells by using the RNeasy Mini Kit (Qiagen, Chatsworth, CA) according to the manufacturer's instructions. Reverse transcription and PCR amplification were carried out under standard conditions by using influenza-specific primers (*16**,**17*). PCR products were purified by using a QIAquick PCR purification kit (Qiagen). Sequencing reactions were performed at the Hartwell Center for Bioinformatics and Biotechnology at St. Jude Children's Research Hospital.

### Sequence Analysis

DNA sequences were compiled and edited by using the Lasergene sequence analysis software package (DNASTAR, Madison, WI). Alignment of each influenza virus sequence was created by using the program Clustal_X (*18**,**19*). Rooted phylograms were prepared by using the neighbor-joining algorithm and then plotted by using NJplot (*20*). In this study, we used the following regions for phylogenetic analyses: PB2, 10-1262; PB1, 66-1368; PA, 8-1290; HA, 1-1002; NP, 55-962; NA, 41-1123; M, 1-889; and NS, 1-842.

### Animal Experiments

Each virus was passaged in 10-day-old embryonated chickens eggs before infection of animals. Virus replication in chickens (specific pathogen-free white leghorn broilers, Charles River SPAFAS), quail (*Coturnix coturnix*, B&D Game Farm), turkey (commercial breeding operation), mice (Balb/C, Jackson Laboratories), and outbred pigs (Midwest Research Swine) was measured after intranasal inoculation with virus-infected allantoic fluid containing 10^6^ EID_50_ (egg infectious dose 50) of virus, as previously described (*21*). All animals were 4–5 weeks old, and the numbers of each species used are listed in Table 1. Tracheal swabs were collected from chickens, quails, and turkeys on postinfection days 3–7, and virus was titrated in 10-day-old embryonated chicken eggs. Mice were weighed daily on days 0–7 postinfection, and animals were killed on day 3, 5, and 7 postinfection, and virus in lung tissue was titrated. Four-week-old pigs free of influenza antibody were intranasally infected with each virus (two pigs for each virus), and nasal swabs were collected on days 2–7 postinfection for virus titration in embryonated eggs. On days 5 and 7 postinfection, one of the two pigs infected with each virus was killed and subjected to pathologic examination and titration of virus in organs.

**Table 1:** Replication of turkey isolates and swine influenza viruses in various animals^a^

Animal	Isolate							
	A/Tk/MN/764/03		A/Tk/NC/16108/03		A/Sw/TX/4199-2/98		A/Sw/NC/29974/02	
	Positive/total^b^	log_10_ EID_50_^c^	Positive/total^b^	log_10_ EID_50_^c^	Positve/total^b^	log_10_ EID_50_^c^	Positve/total^b^	log_10_ EID_50_^c^
Mouse	6/6	4.3 (0.3)	6/6	4.7 (0.3)	6/6	5.3 (0.3)	6/6	5.0 (0.5)
Swine	2/2	2.7 (0.3)	2/2	3.3 (0.3)	2/2	3.7 (0.3)	NT	NT
Turkey	5/5	2.3 (0.3)	3/4	2.3 (0.3)	0/5	–	1/4	1.3 (0.0)
Quail	5/6	3.0 (0.5)	4/6	3.3 (0.5)	0/6	–	0/6	–
Chicken	0/6	–	0/6	–	0/6	–	0/6	–

^a^ NT, not tested; –; negative ^b^Number of animals with a virus-positive lung homogenate (mice) or nasal swab at 3 days postinfection per number of animals infected. ^c^Average log_10_ EID_50_ per milliliter of homogenate (standard deviation).

## Results

### Virus Isolation

Although not always present, clinical signs among turkeys at each farm included depression, coughing, sneezing, loss of appetite, and decreased egg production. Because of the susceptibility of turkeys to H1N1 swine influenza viruses (*22*), the flocks had been vaccinated with an autogenous vaccine against the H1N1 subtype. Four influenza viruses were isolated (in MDCK cells) from tracheal swabs of the affected birds. Only one isolate from each farm was selected for characterization, because preliminary antigenic and sequence analyses of the HA genes of isolates showed that all viruses in each farm were identical. These isolates were designated A/turkey/North Carolina/16108/03 (A/Tk/NC/16108/03) and A/turkey/Minnesota/764/03 (A/Tk/MN/764/03).

### Antigenic and Genetic Characterization

The isolated viruses did not react with any of the reference antisera, even with early (1998) swine influenza virus antisera. We identified them as H3N2 viruses by using RT-PCR under conditions optimized for swine influenza viruses (*16*).

Sequence analysis of the PCR products demonstrated >98% nucleotide identity in each gene segment of the two isolates. According to the Influenza Sequence Database (*23*), the greatest sequence similarity was to genes of recent, triple-reassortant swine H3N2 (viruses containing gene segments derived from swine, avian, and human viruses) and swine H1N2 viruses from the United States (Table 2). A degree of similarity of >97% between the turkey and swine viruses indicated that interspecies transfer had occurred. This report is, to our knowledge, the first of transmission of swine H3N2 viruses to turkeys.

**Table 2:** Type A influenza viruses with the highest nucleotide sequence identity to the turkey isolates

Gene	Nucleotide sequence identity (%)	Virus^a^	Subtype	Reference
HA	97.6	A/swine/NC/29974/02	H3N2	This report
NA	98	A/swine/NC/29974/02	H3N2	This report
PB2	98	A/swine/OH/891/01	H1N2	(*11*)
PB1	98	A/swine/MN/593/99	H3N2	(*7*)
PA	97	A/swine/NE/209/98	H3N2	(*7*)
NP	98	A/swine/OH/891/01	H1N2	(*11*)
M	98	A/swine/MN/34893/01	H1N2	(*10*)
NS	99	A/swine/IN/14810-T/01	H1N2	(*10*)

^a^Named with standard abbreviations of states in the United States.

Phylogenetic analysis of the HA and NA genes of the turkey viruses with those of a number of swine viruses collected in surveillance studies (unpub. data) demonstrated that A/Tk/NC/16108/03 and A/Tk/MN/764/03 are closely related to a swine H3N2 virus isolated in North Carolina in 2002 (A/swine/North Carolina/29974/02) (Figure). The GenBank numbers assigned to the sequences determined in this study are AY779253–AY779270.

**Figure:**
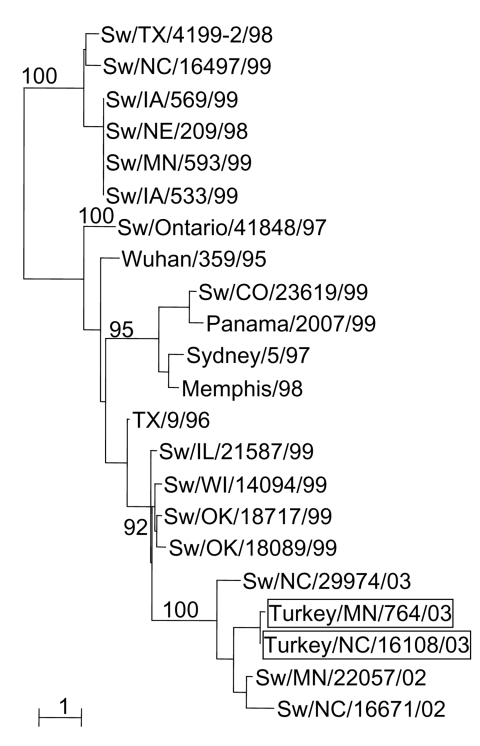
Phylogenetic tree representing the HA nucleotide sequences of two H3N2 influenza viruses isolated from turkeys on geographically distant farms in the United States and of selected swine influenza virus strains. Nucleotide sequences were aligned by using the Clustal_X (*19*) program, and phylograms were generated by the neighbor-joining method using the NJplot program (*20*). The scale is proportional to the numbers of substitutions per nucleotide.

### Animal Infections

Serum taken from each of the 12 healthy birds in one of the infected flocks was highly reactive with the turkey H3N2 isolates (HI titer 1,280), demonstrating that transmission had occurred not only between pigs and turkey but also between turkeys. Therefore, these viruses may have had an opportunity to adapt to the avian host. To investigate this possibility, we compared replication of the two turkey isolates with that of A/swine/NC/29974/02 (the most genetically similar swine virus) and that of A/swine/TX/4199-2/98 (the index triple-reassortant H3N2 virus) in chickens, quail, turkey, mice, and pigs. Animals were infected intranasally with 10^6^ EID_50_ of the respective influenza viruses, and virus was titered in lung homogenates and/or nasal swabs through 7 days after infection (Table 1). All viruses were recovered from pig and mouse lung homogenates (2 –5 log_10_ EID_50_/gram of tissue); this titer demonstrated that viruses of the swine H3N2 triple-reassortant lineage could replicate in pigs and mice without adaptation. None of the viruses were recovered from infected chickens. Only the turkey isolates were reproducibly recovered from turkey and quail. A/swine/NC/29974/02, the most genetically similar swine virus, was isolated only from one of four infected turkeys on day 3 postinfection. Therefore, the turkey isolates appear to be better adapted to avian hosts than do their closest swine counterparts, but certain swine H3N2 viruses also have the potential to replicate in turkeys.

## Discussion

We present the isolation of swinelike H3N2 influenza viruses from two geographically distinct turkey farms in the United States. These viruses are closely related to swine H3N2 viruses that emerged in pigs in the United States in 1998 and have since become endemic. Although infection of turkeys with H1N1 and H1N2 swine influenza viruses has been documented (*13**,**22**,**24*), to our knowledge, this report is the first of swinelike H3N2 infection in this host. The clinical signs of infection in these turkeys were not severe, but our findings have implications for influenza ecology and the possibility of further evolution of these viruses.

Two properties of the swine-like H3N2 infection in turkeys raise concerns about the potential for further viral evolution. The first is that the H3N2 viruses have been successfully established in pigs and have demonstrated an ability to reassort with human (*8*), swine (*11*), and avian viruses (*10*). The second is that turkeys appear to be highly susceptible to infection by influenza viruses from aquatic birds (*25*), thus increasing opportunities for further reassortment. Although the fitness of such a swine-avian reassortant cannot be predicted, and the infected flocks showed no evidence of coinfection with avian viruses, continuing to monitor turkey populations for the presence of swine virus-like gene segments is prudent.

We found strong evidence that turkey-to-turkey transmission had occurred in at least one of the flocks, and the viruses were similar despite their geographically distinct origins. Although detailed information is not available, there are no obvious links between the infected flocks, and direct movement of turkeys between the two farms was unlikely. Further epidemiologic investigation of the flocks and surrounding swine herds is warranted. In a small scale retrospective screen of 125 turkey serum samples collected from 2000 to 2004, we were unable to detect any evidence of H3N2 infection using standard HI assays (data not shown), which suggests that such infections are not widespread. Although the most obvious explanation for the dual outbreak is a common source of infection, both flocks may have been infected by a swine virus circulating in pigs in both areas.

Although we recovered one swine isolate genetically similar to the turkey isolates from one of 4 infected turkeys, the ability of only the turkey isolates to replicate in quails suggests that the viruses may have already begun to adapt to the avian host. This adaptation to an avian host did not occur at the expense of the ability to replicate in pigs. Both turkey isolates were shed by experimentally infected swine for at least 5 days; this shedding pattern is consistent with that of genetically similar swine isolates. To speculate that the turkey isolates can replicate in quail and, possibly, other avian hosts as a result of their passage in turkeys is tempting. However, the traits that allowed the viruses to be transmitted to turkeys may also allow them to infect quail. The adaptation did not appear to be a result of egg passage, as we found no differences in HA sequence between MDCK and egg-grown virus. To investigate the possible mechanisms of avian adaptation, we compared the amino acid sequences of the HA1 proteins of the turkey isolates and the swine H3N2 viruses. The turkey isolates and A/swine/NC/29974/02 differed at only seven amino acids, two of which (residues 137 and 226) were in the receptor binding site. Although we found substitutions at these two sites in other swine isolates represented in sequence databases (data not shown), only the turkey isolates had both the Y137S and V226I substitutions. Ongoing reverse genetics experiments will help to identify the relative contributions of these amino acids to avian adaptation.

The introduction of H3N2 viruses to the U.S. swine population has significantly affected patterns of animal influenza in this country. Not only have the H3N2 viruses successfully established and reassorted in pigs, but H1N1 (*22*), H1N2 (*13*), and now H3N2 isolates carrying the internal genes of H3N2 viruses have been isolated from birds. Viruses of this genetic composition will likely continue to evolve and cause problems for animal and, potentially, human health. The swine population is likely a reservoir of yet another lineage of influenza viruses that have demonstrated the ability to reassort and be transmitted between species.

Repeated introductions of swine influenza viruses to turkeys, which may be coinfected with avian influenza viruses, provide opportunities for the emergence of novel reassortants with genes adapted for replication in pigs or even humans. Our studies emphasize the continuing need to monitor pigs and domestic birds to better understand interspecies transmission and the emergence of novel influenza viruses that have the potential to infect humans.
